# Pharmacokinetics, Pharmacodynamics and Antiviral Efficacy of the MEK Inhibitor Zapnometinib in Animal Models and in Humans

**DOI:** 10.3389/fphar.2022.893635

**Published:** 2022-06-15

**Authors:** Julia Koch-Heier, Annika Schönsiegel, Lara Maria Waidele, Julian Volk, Yvonne Füll, Christian Wallasch, Sebastian Canisius, Michael Burnet, Oliver Planz

**Affiliations:** ^1^ Department of Immunology, Interfaculty Institute for Cell Biology, Eberhard Karls University Tuebingen, Tuebingen, Germany; ^2^ Atriva Therapeutics GmbH, Tuebingen, Germany; ^3^ Synovo GmbH, Tuebingen, Germany

**Keywords:** MEK inhibitor, pharmacokinetic, pharmacodynamic, antiviral therapy, zapnometinib, WES

## Abstract

The mitogen-activated protein kinase (MEK) inhibitor zapnometinib is in development to treat acute viral infections like COVID-19 and influenza. While the antiviral efficacy of zapnometinib is well documented, further data on target engagement/pharmacodynamics (PD) and pharmacokinetics (PK) are needed. Here, we report zapnometinib PK and PD parameters in mice, hamsters, dogs, and healthy human volunteers. Mice received 25 mg/kg/day zapnometinib (12.5 mg/kg p. o. twice daily, 8 h interval). Syrian hamsters received 30 mg/kg (15 mg/kg twice daily) or 60 mg/kg/day once daily. Beagle dogs were administered 300 mg/kg/day, and healthy human volunteers were administered 100, 300, 600 and 900 mg zapnometinib (once daily p. o.). Regardless of species or formulation, zapnometinib maximum plasma concentration (C_max_) was reached between 2–4 h after administration with an elimination half-life of 4–5 h in dogs, 8 h in mice or hamsters and 19 h in human subjects. Doses were sufficient to cause up to 80% MEK inhibition. Across all species approximately 10 μg/ml zapnometinib was appropriate to inhibit 50% of peripheral blood mononuclear cells (PBMC) MEK activity. In mice, a 50%–80% reduction of MEK activity was sufficient to reduce influenza virus titer in the lungs by more than 90%. In general, while >50% MEK inhibition was reached *in vivo* at most doses, 80% inhibition in PBMCs required significantly higher doses and appeared to be the practical maximal level obtained *in vivo*. However, the period of reduced phosphorylated extracellular-signal regulated kinase (pERK), a measure of MEK inhibition, was maintained even after elimination of zapnometinib from plasma, suggesting a sustained effect on MEK consistent with regulatory effects or a slow off-rate. These data suggest a target plasma C_max_ of at least 10 μg/ml zapnometinib in further clinical studies.

## Introduction

The coronavirus disease 2019 (COVID-19) pandemic and the influenza pandemic in 2009 have renewed interest in therapeutics for respiratory viruses (Monto et al., 2011; Robertson and Inglis, 2011; Mackey and Liang, 2012; Huff and Singh, 2020; Lombardo et al., 2021). As demonstrated in a range of infections (Human immunodeficiency virus (HIV), Herpes, hepatitis C), antiviral and anti-inflammatory therapies (usually corticosteroids—e.g., ocular keratitis) are useful stop-gaps or treatments for viral infections while vaccines are in development or when immune evasion overcomes vaccine effect. However, respiratory viruses present challenges not apparent in systemic virus types and both COVID-19 and influenza disease course show that treatment initiation after symptom onset usually misses peak viral replication and thus limits the immediate utility of direct antivirals (see requirements for early application of Oseltamivir) (Ison, 2013). In severe stages of disease, characterized by an extreme inflammatory response, there is significant extant viremia, and the objectives of therapy are both to contain inflammation and reduce viral replication (Kirtipal et al., 2020; Muralidar et al., 2020; Asselah et al., 2021). At this point, immunomodulatory treatments like dexamethasone have been employed to limit severe signs (Selvaraj et al., 2020; Sun et al., 2020; Horby et al., 2021). While corticosteroids manage viral induced inflammation well, they simultaneously enhance viremia which can be detrimental (in the first severe acute respiratory syndrome coronavirus 2 (SARS-CoV-2) outbreak where dexamethasone reduced signs but not mortality). Direct antiviral drugs, especially in monotherapy, exert very strong selection for resistance. This is already apparent for Neuraminidase inhibitors and was a major issue in HIV management prior to the implementation of combination therapy (Smith et al., 2002; McKimm-Breschkin et al., 2007; Rameix-Welti et al., 2008; Meijer et al., 2009; Jackson et al., 2011; Shirley, 2020). Thus, an ideal agent would be one that could reduce viremia while also managing inflammation, being less prone to resistance selection and being useful for more than one strain or type of virus.

A new approach for antiviral therapy is the inhibition of host cell factors that viruses require for replication. This could overcome the current limitations of antivirals and may also circumvent the problems of resistance development (Gong et al., 2009; Ludwig, 2009). Signaling pathways such as the Raf/MEK/ERK cascade are relevant for antiviral interventions since activation of this signaling cascade is required by a large variety of RNA viruses. The activation then leads to stepwise phosphorylation and activation of the serine-threonine kinase Raf, which in turn phosphorylates and activates the dual-specificity protein kinases MEK1 and MEK2. MEK catalyzes the phosphorylation and activation of its only known substrates, the mitogen-activated protein (MAP) effector kinases ERK1 and ERK2 (Sebolt-Leopold, 2004; Yoon and Seger, 2006; Montagut and Settleman, 2009; Wei et al., 2016; Eblen, 2018). Phosphorylated ERK1/2 (pERK1/2) finally activates various cellular functions such as cell proliferation, differentiation, apoptosis, cytokine production, and immune responses (Molina and Adjei, 2006; Li et al., 2016). Several RNA viruses such as influenza A and B viruses, respiratory syncytial virus (RSV), Hantavirus, Dengue virus, hepatitis C virus and SARS corona viruses require an active Raf/MEK/ERK signaling cascade for assembly and replication. Inhibition of the cascade using MEK inhibitors prevents functional assembly and leads to reduced viral load (Planz et al., 2001; Pleschka et al., 2001; Dong et al., 2002; Ludwig et al., 2004; Olschläger et al., 2004; Pleschka, 2008; Ludwig, 2009; Shyu et al., 2010; Droebner et al., 2011; Pinto et al., 2011; Zhang et al., 2012; Planz, 2013; Kumar et al., 2018; Preugschas et al., 2019; de Oliveira et al., 2020; Furuyama et al., 2021; Valencia et al., 2021).

In addition to supporting intracellular propagation of the virus, the Raf/MEK/ERK signaling pathway is involved in many processes of the innate and adaptive immune response (Dong et al., 2002; Arthur and Ley, 2013). Inhibition of the pathway leads to a reduced hypercytokinemia (cytokine storm), a shift from a T_H_2 to a T_H_1 immune response, reduction of regulatory T cell (T_reg_) response and a prolonged clonal expansion of T cells (Nakayama and Yamashita, 2010; Lieske et al., 2015; Ebert et al., 2016). All these actions support an effective cellular immune response against the virus. Therefore, MEK inhibition shows a dual effect in viral infections where disease signs are dominated by inflammation; it reduces the viral load and modulates the immune response toward adaptive responses (Laure et al., 2020). One promising candidate MEK inhibitor is zapnometinib (ATR-002, PD0184264), which is the active metabolite of CI-1040, a drug that was originally developed for cancer treatment (Sebolt-Leopold et al., 1999; Lorusso et al., 2005). In a previous study, the antiviral efficacy of zapnometinib against influenza A and B viruses was already determined *in vitro* with an EC_50_ of 4.2–6.4 µM (Laure et al., 2020). Furthermore, Laure and others confirmed the antiviral efficacy *in vivo*, determined the cytotoxicity in human PBMCs (CC_50_ = 321.5 µM) and in other cell lines (Laure et al., 2020). In addition, zapnometinib also exhibits potent anti-SARS-CoV-2 activity as evidenced by a reduction in viral growth in cell lines (Schreiber et al., 2022). Thereupon, zapnometinib has entered a phase 2 clinical trial for treatment of hospitalized COVID-19 patients (NCT04776044).

To support dose optimization, we investigated pharmacokinetic (PK) and pharmacodynamic (PD) parameters in mice, hamsters, dogs, and healthy human volunteers. Our goal was to establish the degree of MEK inhibition over time following various peak plasma exposures and to associate this with apparent antiviral activity in influenza virus infected mice. These data support zapnometinib dose selection in future clinical trials for antiviral efficacy.

## Materials and Methods

### Cells and Virus

Madin-Darby canine kidney cells (MDCK II, ATCC^®^, CRL-2936™) for the determination of viral titers in foci assays were purchased from ATCC and cultured in Iscove’s Modified Dulbecco’s Medium (IMDM, Thermo Fisher Scientific, Waltham, Massachusetts, MA, United States) supplemented with 10% (v/v) fetal bovine serum (FBS, Sigma-Aldrich, St. Louis, Missouri, MO, United States), 100 U/mL penicillin and 100 μg/ml streptomycin (Sigma-Aldrich, St. Louis, Missouri, MO, United States). Cells were maintained in a 37°C and 5% CO_2_ atmosphere. Human peripheral blood mononuclear cells (PBMCs) for the half maximal inhibitory concentration (IC_50_) determination were isolated from healthy volunteers registered with the Biobank of the Department of Immunology at the University of Tuebingen. Ethical approval was obtained upon review by the Ethics Board of the Medical Faculty of the Eberhard Karls University Tuebingen and the University Hospital Tuebingen (887/2020BO2). For the virus inhibition experiment influenza A virus (IAV) strain A/Regensburg/D6/09 (H1N1pdm09) was used. The virus was provided by the Robert-Koch Institute of Berlin.

### Animals

In total 15 eight-week-old female C57BL/6 mice (Charles River Laboratories, Sulzfeld, Germany) with a body weight of 21–24 g at administration were used for the antiviral and pharmacokinetic study (*n*_Control_ = 3; *n*_Zapnometinib_ = 12). The animals were fed with standard food. Drinking water was available *ad libitum*. The mice were housed in type 2 cages with four animals per cage under BSL-II conditions. During this study, assessments included mortality checks and body weight. Mice were sacrificed 24 h post infection. The mouse study was reviewed and approved by the Regional Council Tuebingen, registered under number IM01-13. In total, 10 eight-week-old male Syrian hamster (Janvier, France) with a body weight of 86–102 g at administration were used for the pharmacokinetic and pharmacodynamic study (*n*_30 mg/kg_ = 5; *n*_60 mg/kg_ = 5). The hamster were sacrificed 24 h post the first treatment. The animals were fed with standard diet. Drinking water was available *ad libitum*. The hamster animal experiments were carried out at Synovo GmbH (Tuebingen, Germany). The hamster study was reviewed and approved by the Regional Council Tuebingen, registered under number SYN 03/21–0001. In total, 10- to 24-months old beagle dogs (Marshall Bioresources, North Rose, NY 14516, United States) with a body weight of 6.6–9.2 kg at the start of treatment were used for the pharmacokinetic and pharmacodynamic study. Each dog received each formulation with a dosing holiday of at least 2 days between occasions to allow for washout. The animals were fed with standard food. Drinking water was available *ad libitum*. The animals were group housed in stainless steel dog cages equipped with an automatic watering system. The dog study was performed at Citoxlab North America (Laval, Canada). During this study, assessments included mortality checks, clinical observations (health status, presence of wounds, changes in appetite, feces, skin discoloration), behavioral changes, and body weight. This study was reviewed and approved by the Institutional Animal Care and Use Committee (IACUC) and registered under the study number 2018–1,172. The animals were returned to the Citoxlab spare colony after the last blood sample collection.

### Drugs and Dosing Administration

Zapnometinib [2-(2-Chloro-4-Iodophenylamino)-N-3,4-Difluoro benzoic acid] (M = 409.56 g/mol) was synthesized at ChemCon GmbH (Freiburg, Germany). The doses used in this study were chosen based on previously unpublished preclinical data. In mice, a dose of 75 mg/kg zapnometinib was well tolerated. The toxicity of zapnometinib was evaluated in single- and repeat-dose p. o. studies of up to 1-month duration in rats and dogs. The genetic toxicity of zapnometinib was investigated in compliance with ICH S2; a standard battery of genotoxicity studies was performed, comprising an Ames test, an *in vitro* chromosome aberration test in human lymphocytes and an *in vivo* micronucleus assay in rats. Thus, the doses used in this manuscript were safe (Koch-Heier et al., manuscript in preparation). For treatment of mice, 10.5 mg zapnometinib were dissolved in 5% (v/v) dimethyl sulfoxide (DMSO) (Carl Roth GmbH, Karlsruhe, Germany) and further diluted with 15% (v/v) Cremophor EL (Merck KGaA, Darmstadt, Germany), and 80% (v/v) phosphate-buffered saline (PBS) (Gibco, Carlsbad, CA, California, United States). Each mouse received a dose of 25 mg/kg zapnometinib in an application volume of 200 µL of the formulation by oral gavage. The first treatment was given on day 0 (1 h prior to influenza virus infection) and the second treatment 7 h post infection. The liquid dose formulation for the hamster study was prepared as two-times concentrated stock solution of zapnometinib in 5% (v/v) DMSO, 30% (v/v) polyethylene glycol (PEG) 400, 20% (w/v) Glucose, 10% (v/v) cyclodextrin as Captisol^®^ (stock was 40% (w/v) in water (H_2_O)). The 2x stock solution was then further diluted in strawberry flavored syrup and administered orally using a voluntary procedure. Each hamster received either a dose of 60 mg/kg once daily (OD) or a dose of 15 mg/kg zapnometinib twice daily (BID). The application volume of the zapnometinib formulation was 2 ml/kg for the hamster on day 0, and the second administration occurred 12 h after the first dose. For treatment of dogs, the liquid dose formulation (formulation 5) was prepared to a concentration of 10 mg/ml. Zapnometinib was dissolved in 5% (v/v) DMSO. Then 30% (v/v) of PEG 400 was added and stirred until homogenous. This was followed by the addition of an appropriate volume of 40% (w/v) Captisol^®^ to obtain a final concentration of 7.4% (w/v) and stirred until homogenous. The pH was adjusted with sodium hydroxide and hydrochloric acid to a final pH of 7.4. The formulation was stored refrigerated (set to maintain at 2–8°C) pending use for dosing. Each dog received a dose of 30 mg/kg zapnometinib. The capsule formulation (formulation 1) contained a selected hot melt extrusion (HME) formulation (150 mg zapnometinib/capsule). The tablet formulation either contained micronized zapnometinib, coated for moisture/light protection, without enteric functionality (formulation 2), micronized zapnometinib, coated for enteric functionality (formulation 3) or non-micronized zapnometinib, coated for moisture/light protection without enteric protection functionality (formulation 4). Each tablet contained 50 mg zapnometinib. Each dog received 2 capsules for a total dose of 300 mg zapnometinib on day 1 or 6 tablets for a total dose of 300 mg zapnometinib on days 4, 7, and 11 as per schedule presented in Table 1. For treatment in humans, zapnometinib was formulated as a tablet with 100 mg zapnometinib per tablet in accordance with Good Manufacturing Practice (GMP) as required by the current good clinical practice (GCP) and administered to the subjects regardless of body weight (b.w.). Each volunteer received once daily a dose of 100 mg up to 900 mg zapnometinib in the single ascending dose (SAD) part, and up to 600 mg for 7 days in the multiple ascending dose (MAD) part.

**TABLE 1:** Overview of zapnometinib formulations used in the beagle dog study.

Formulation	Dosing day	Dosing route	Dose level (mg/animal/occasion)^e^ mg/kg^f^	Number of animals	
				M	F
1	1	Oral (capsule)^a^	300^e^	5	5
2	4	Oral (tablet)^b^	300^e^	5	5
3	7	Oral (tablet)^c^	300^e^	5	5
4	11	Oral (tablet)^d^	300^e^	5	5
5	20	Oral (liquid)	30^f^	5	5

a Capsules containing best performing selected hot melt extrusion (HME) formulation (150 mg zapnometinib/capsule).

b Tablets containing micronized zapnometinib, coated for moisture/light protection, without enteric functionality (50 mg zapnometinib/tablet).

c Tablets containing micronized zapnometinib, coated for enteric functionality (50 mg zapnometinib/tablet).

d Tablets containing non-micronized zapnometinib, coated for moisture/light protection without enteric functionality (50 mg zapnometinib/tablet).

e Units for oral (capsule/tablet) administration.

f Units for oral (gavage) administration. **M**—Male; **F**—Female.

### Study Design and Participants of the Human Phase 1 Clinical Trial

Healthy men and women between 18 and 55 years of age (inclusive), who weighed at least 50 kg with a body mass index (BMI) between 18.0 and 31.0 kg/m^2^ (inclusive) were enrolled in this study. Exclusion criteria were clinically significant illnesses, positive urine test for selected drugs of abuse, positive alcohol breath test at screening and upon check-in at the clinical site, positive hepatitis panel (including hepatitis B surface antigen [HBsAg] and anti-hepatitis C virus [HCV] antibodies), and positive screens for human immunodeficiency virus (HIV) antibody. This Phase 1 study was a randomized, double-blind, placebo-controlled study (clinicaltrials.gov: NCT04385420) consisting of two parts. Part 1 was a SAD study with 40 healthy volunteers who were randomized to active or placebo treatment in a 4:1 ratio (4 cohorts with *n*_(*Zapnometinib*)_ = 8; *n*_(*placebo*)_ = 2 in each cohort). Subjects receiving active treatment were treated with either 100 mg, 300 mg, 600 mg, or 900 mg zapnometinib. Part 2 was a MAD study with 30 healthy volunteers in three cohorts with the same randomization as in the SAD part (3 cohorts with *n*_(*Zapnometinib*)_ = 8; *n*_(*placebo*)_ = 2 in each cohort). Each subject in the MAD cohorts received doses of zapnometinib of either 100 mg, 300 mg, or 600 mg once daily for seven days, or placebo. The safety assessment in this study included monitoring of adverse events (AEs), clinical laboratory tests, electrocardiograms (ECG), vital signs, and physical examination. The study protocol and its amendments were reviewed by the Commissie voor Medische Ethiek ZNA, Antwerp, Belgium. This study was conducted in accordance with the ethical principles that have their origin in the Declaration of Helsinki and the International Council for Harmonization of Technical Requirements for Pharmaceuticals for Human Use (ICH) Note for Guidance on Good Clinical Practice (GCP) (CPMP/ICH/135/95) and with applicable local requirements.

### Infection of Mice With Influenza A Virus

Mice were anesthetized by intraperitoneal (i.p.) injection of ketamine (10 mg/kg) solution and then infected intranasally with 1.5 × 10^5^ foci forming units (ffu) (5 x median lethal dose, MLD_50_) influenza A virus (strain: A/Regensburg/D6/2009 H1N1pdm09) in 50 µl PBS by inoculating 25 µL into each nostril.

### Blood Sampling and Preparation of Plasma Samples

For the study in mice, 30 µl blood samples were collected in microvettes containing lithium heparin as anticoagulant (Sarstedt, Nümbrecht, Germany) at the following timepoints: pre-dose, 30 min, 1, 2, 4, 6, 8, 8.5, 9, 10, 22, and 24 h after treatment. The samples were kept on wet ice pending centrifugation. In the hamster study, blood samples of 50 µl were collected using heparinized capillaries at pre-dose, 30 min, 1, 2, 4, 8, 12, and 24 h after treatment. The samples were kept on wet ice pending centrifugation. In the dog study, 1 ml blood samples were collected into tubes containing K_2_-EDTA as anticoagulant at the following timepoints: pre-dose, 30 min, 1, 2, 4, 6, 9, 12, 15, and 24 h after treatment. The samples were kept on wet ice pending centrifugation. All samples were centrifuged at 1,500 x g at 4°C for 10 min. Plasma was collected and stored at −20°C or on wet ice until further analysis. The remaining blood pellet was used for isolation of peripheral blood mononuclear cells (PBMCs). For the study in human healthy volunteers 8 ml venous blood samples were collected in cellular preparation tubes (CPT^TM^ tubes) containing lithium heparin as anticoagulant (BD Biosciences, Heidelberg, Germany) at pre-dose, 30 min, 1, 2, 4, 8, 12, and 24 h post dose on day 1, and at pre-dose on day 2–4 (MAD part only). The samples were centrifuged at 1,650 x g at room temperature (RT) for 20 min. Plasma was collected and stored at −20°C until further analysis. The remaining blood pellet was used for isolation of PBMCs.

### Pharmacokinetic Analysis

Pharmacokinetic analysis of the mouse plasma samples was performed using standard procedures at Prolytic GmbH (Frankfurt, Germany). Hamster plasma samples were analyzed at Synovo GmbH (Tuebingen, Germany) using HPLC MS/MS (Agilent 1260 HPLC with Sciex API4500) with linear response for plasma concentrations between 15 nM and 33 µM (6.1 ng/ml to 13.5 μg/ml). Pharmacokinetic analyses of the dog plasma samples were performed at Pharmascience Inc. (Montreal, Canada) using an HPLC MS/MS method for the determination of zapnometinib plasma concentrations. The validated calibration range for this assay is from 0.6 μg/ml to 500 mg/ml for which all validation requirements were successfully evaluated. Regressions, calibration standard, quality control sample, animal sample result tables, acquisition methods and chromatograms were generated by Applied Biosystems Analyst^TM^ software, version 1.6.2. Plasma concentrations of zapnometinib of the human samples were determined at SGS Life Sciences (Bioanalysis Department, Wavre, Belgium) using a validated HPLC MS/MS method with a lower limit of quantification of 10 ng/ml for the analyte zapnometinib. Statistical analysis was performed by SGS (demography and safety analyses, exposure, and compliance) using SAS® version 9.4 (SAS Institute Inc., Cary, NC, United States).

### Isolation of Peripheral Blood Mononuclear Cells

PBMCs were isolated from the same blood samples (30 µl mouse blood, 50 µl hamster blood, or 1 ml dog blood) used for pharmacokinetic analysis. After plasma separation as described in the section above (Blood sampling and preparation of plasma samples), the remaining red blood cells were lysed by adding 1 volume of red blood cell (RBC) lysis buffer (Thermo Fisher Scientific, Waltham, MA, United States) (300 µl for mice, 500 µl for hamster, and 10 ml for dogs). The samples were incubated for 15 min at RT and centrifuged at 500 x g for 5 min (RT). The supernatant was removed completely, and the pellet was washed twice with PBS. The PBMC cell pellets were store at −80°C until further analysis. PBMCs of 8 ml human blood were isolated using CPT^TM^ tubes. After a 20-min centrifugation at 1,650 x g at room temperature, the plasma was collected for PK analysis as described in the section above (Blood sampling and preparation of plasma samples). The PBMC layer was collected in a 15 ml tube (Greiner Bio-One, Frickenhausen, Germany), and washed twice with PBS by centrifugation at 300 x g for 10 min at RT. The PBMC pellets were store at −80°C until further PD assessment.

### Pharmacodynamic Assessment to Determine Level of Mitogen-Activated Protein Kinase Inhibition

The pharmacodynamic assessment was performed using the Wes^™^ (Simple Western^™^) system which uses capillary electrophoresis to separate, identify and quantify proteins of interest. A chemiluminescence signal for each identified protein is detected and the area under the peak is calculated, which is then used for quantification. The 0.1x Sample Buffer, 400 mM dithiothreitol (DTT) solution, 5x Fluorescence Master Mix, and the Biotinylated Ladder (ProteinSimple^®^, Abingdon, Oxford, United Kingdom) were prepared according to manufacturer’s instructions. For all species, PBMCs were lysed in 1x radioimmunoprecipitation assay (RIPA) buffer (containing 0.24% (w/v) tris base (Sigma-Aldrich, St. Louis, MO, United States), 0.88% (w/v) NaCl (Carl Roth GmbH, Karlsruhe, Germany), 0.2% (v/v) 500 mM EDTA (Sigma-Aldrich), 1% (v/v) Triton X-100 (Sigma-Aldrich), 0.5% (w/v) sodium deoxycholate (Sigma-Aldrich), 0.1% (w/v) sodium dodecyl sulfate (SDS) (Carl Roth), 10% (v/v) glycerol (Carl Roth), 0.05% phenylmethyl sulfonyl fluoride (PMSF) (Carl Roth), 0.01% Benzonase^®^ Nuclease (Sigma-Aldrich), protease inhibitor cocktail (Roche, Basel, Switzerland), and phosphatase inhibitor cocktail (Roche) in H_2_O), further diluted with 0.1x Sample Buffer (ProteinSimple^®^, Abingdon, Oxford, United Kingdom) to adjust the protein concentration to 1 μg/μl. Then the 5x Fluorescence Master Mix in DTT (ProteinSimple^®^) was added, and the samples were denatured for 5 min at 95°C, loaded onto the assay plate and analyzed using specific antibodies. The primary antibodies for phospho ERK1/2 (Phospho-p44/42 MAPK (Erk1/2) (Thr202/Tyr204) (D13.14.4E) XP^®^ Rabbit mAb, Cat# 4370) and ERK1/2 (p44/42 MAPK (Erk1/2) (137F5) Rabbit mAb, Cat# 4695) were purchased from Cell Signaling Technology (Danvers, MA, United States) and used at a 1:50 (pERK1/2) or 1:100 (ERK1/2) dilution in Antibody Diluent (ProteinSimple^®^). The anti-rabbit secondary antibody (Cat# DM-001) was also purchased from ProteinSimple^®^ and was ready to use. Data were analyzed with Compass software for simple western (ProteinSimple^®^). Using the ratio of pERK to ERK of each sample compared to control (pre-dose), the level of ERK phosphorylation was calculated and then converted into the percentage level of MEK inhibition.

### Determination of Viral Load in Mouse Lungs

Mice were sacrificed 24 h after zapnometinib treatment. Lungs were removed, weighed, and transferred into a Lysing Matrix D tube (MP Biomedicals, Germany). 500 µL ice-cold PBS was added, the lungs were homogenized using a FastPrep FP 120 (Savant) and centrifuged for 10 min at 18,000 x g at 4°C. The supernatant was used to perform an AVICEL^®^ foci assay on MDCK II cells as previously described (Matrosovich et al., 2006; Haasbach et al., 2011). Briefly, MDCK II cells were seeded in 96-well cell culture plates and incubated overnight at 37°C and 5% CO_2_. The cells were washed with PBS and infected with the lung tissue supernatants in triplicates. 1 h post infection the inocula were removed and the Avicel^®^ overlay was added and incubated for 22 h at 37°C and 5% CO_2_. The cells were fixed with 4% paraformaldehyde (PFA) solution and permeabilized with 0.3% Triton X-100 in PBS. Cells were then immunostained with mouse anti-IAV nucleoprotein monoclonal antibody (Bio-Rad, California, CA, United States) for 60 min at RT, followed by washing and a 30-min incubation with goat anti-mouse IgG-HRP antibody (Jackson ImmunoResearch Laboratories, Pennsylvania, PA, United States). After washing, True Blue^TM^ peroxidase substrate (SeraCare Life Science, Milford, KS, United States) was added for 10 min at RT. Finally, the number of foci were counted to determine the virus titer as foci forming units per ml (ffu/ml).

### Determination of IC_50_ Values in Human Peripheral Blood Mononuclear Cells

Human whole blood was treated with different concentrations (ranging from 0–100 μg/ml) of zapnometinib for 1 h at 37°C and 5% CO_2_. Subsequently, PBMCs were isolated using 1x RBC lysis buffer and lysates were prepared in 1x RIPA buffer for further analysis with Wes^™^ technology. Using the ratio of pERK to ERK of each sample compared to control (DMSO), the level of ERK phosphorylation was calculated. For dose response curves and calculation of IC_50_ values, data were fitted using GraphPad Prism software version 9 (GraphPad Software Inc, San Diego, CA, United States).

### Statistical Analysis

Unless otherwise noted, data were collected in MS Excel, and statistical analyses were performed using GraphPad Prism software version 9 (GraphPad Software Inc). Statistical details for each experiment are described in the corresponding figure legends. Differences in viral titer were analyzed by unpaired *t* test with Welch’s correction. *p* values <0.05 were considered statistically significant.

## Results

### Influence of Zapnometinib on Mitogen-Activated Protein Kinase and Extracellular-Signal Regulated Kinase Phosphorylation

First, we investigated whether binding of zapnometinib to MEK inhibits phosphorylation of MEK and thus also prevents further phosphorylation of ERK. Since ERK is the only substrate downstream of MEK, usually reduction of ERK phosphorylation is measured to determine MEK inhibition. PBMCs from healthy donors were isolated, treated with zapnometinib, stimulated with Phorbol-12-Mystrat-13-Acetat (PMA) and analyzed for the reduction of MEK and ERK phosphorylation. We could show that zapnometinib directly prevents the phosphorylation of MEK and consequently causes inhibition of the phosphorylation of the MEK1/2-dependent kinase ERK1/2 (Supplementary Figure S1). Because ERK phosphorylation is routinely used to study MEK inhibition, we have also focused on it in the following experiments.

### Correlation of Pharmacodynamics, Pharmacokinetics, and Antiviral Efficacy in Mice After Influenza Virus Infection

One major objective of this study was to correlate pharmacokinetic and pharmacodynamic parameters with the antiviral efficacy of zapnometinib *in vivo*. Therefore, we infected mice with a lethal dose of H1N1pdm09 (1.5 × 10^5^ ffu) and treated them orally with 25 mg/kg zapnometinib twice daily, for a total dose of 50 mg/kg/day. The first dose was administered 1 h prior to infection and the second dose 7 h post infection (8 h after the previous dose). Blood samples were collected at different time points (as mentioned in the method section) for pharmacokinetic and pharmacodynamic assessments. Lungs of the sacrificed mice were taken 24 h post infection for the determination of the viral titer.

**FIGURE 1:**
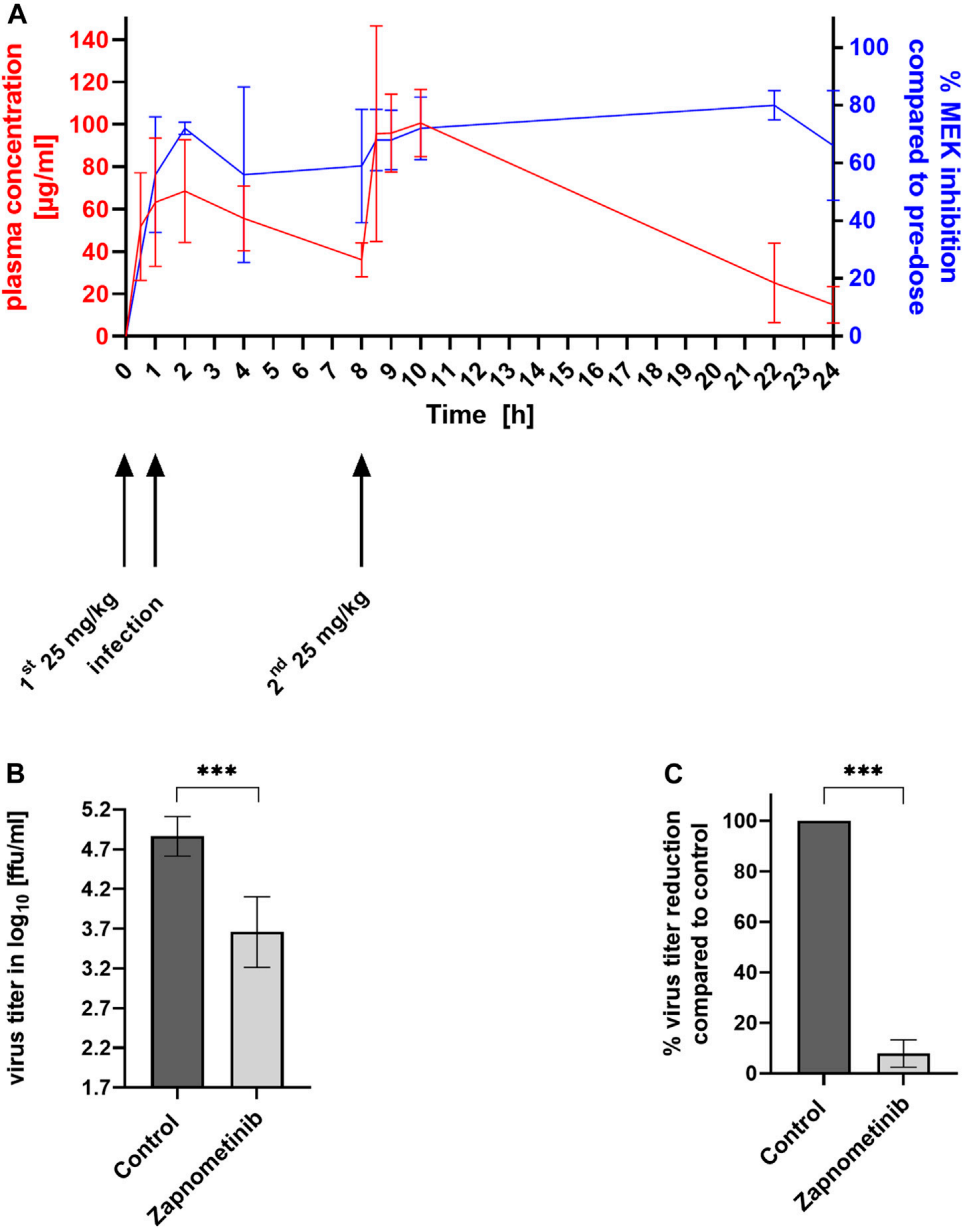
MEK Inhibition, Plasma Concentration and Reduction of Virus Titer in Influenza Virus Infected and Zapnometinib Treated Mice. Female C57BL/6 mice were infected with influenza A virus strain H1N1pdm09 and treated twice with 25 mg/kg zapnometinib by oral gavage 1 h prior and 7 h post infection. Blood was collected at different time points (*t* = 0–24 h) for pharmacokinetic and pharmacodynamic analyses. **(A)** Association between zapnometinib plasma concentration (left *y*-axis, red line) and MEK inhibition (right *y*-axis, blue line). Data presented as mean ± SD **(B,C)** Antiviral efficacy of zapnometinib against H1N1pdm09 in mice. **(B)** Virus titers in the lung of zapnometinib treated mice (*n* = 12) compared to DMSO treated control (*n* = 3) were given as log_10_ ffu/ml. Unpaired *t* test with Welch’s correction was used to test for statistical significance of the difference between control and zapnometinib treated group (***p < 0.001). **(C)** Virus titer in % reduction of zapnometinib treated mice (*n* = 12) compared to DMSO treated control (*n* = 3) Unpaired *t*-test with Welch’s correction was test for statistical significance of the difference between the two groups (***p < 0.001).

First, we investigated the level of MEK inhibition in PBMCs after treatment compared to pre-dose levels. Zapnometinib treatment resulted in MEK inhibition levels between 56 ± 20% to 80 ± 5% as measured by the reduction of ERK phosphorylation. A first maximum of MEK inhibition of 72 ± 10% was reached 2 h after the first treatment and thereafter declined between 59 ± 19% to 56 ± 30% prior to the second treatment (*t* = 8 h). Afterwards the level of MEK inhibition increased by about 10% following increased plasma levels and then steadily climbed to a second maximum of 80 ± 5% MEK inhibition at 22 h after the first treatment (14 h after the second treatment) (blue line, Figure 1A). The pharmacokinetic analysis demonstrated a rapid increase in the plasma concentration of zapnometinib (red line, Figure 1A). The area under the curve (AUC) was calculated to be 1,389 μg/ml*h. Zapnometinib concentrations in the plasma reached a first maximum 2 h after the first treatment (T_max,1_) with a maximum concentration of 69 ± 24 μg/ml (C_max_). Prior to the second treatment (*t* = 8 h) the plasma concentration declined to 36 ± 8 μg/ml followed by an increase to 101 ± 16 μg/ml two hours after the second administration (T_max,2_ = 10 h). At the end of the observation period (*t* = 24 h) the plasma concentration of zapnometinib had decreased to 15 ± 9 μg/ml.

Association of the %MEK inhibition (pharmacodynamics, blue line) with the plasma concentration of zapnometinib (pharmacokinetics, red line) is presented in Figure 1A. MEK inhibition increased with increasing plasma concentration. The level of MEK inhibition remained at high levels, even with decreasing plasma concentrations. At the end of the observation period (*t* = 24 h) 66 ± 19% MEK inhibition was apparent at a plasma concentration of 15 ± 9 μg/ml zapnometinib.

To investigate the impact of 60%–80% MEK inhibition in influenza virus infected mice on the viral load, the animals were sacrificed 24 h after infection and virus titers were determined using a standard virus titration assay. Treatment of mice with 25 mg/kg BID resulted in a significant reduction of virus titer of 1.2 log_10_ ffu/ml (Figure 1B), which corresponds to a 92 ± 1% reduction (Figure 1C) compared to control animals.

The data from the influenza virus mouse model demonstrate that 60% –80% of MEK inhibition are sufficient to significantly reduce the viral load using zapnometinib in a dosage that is well tolerated and not toxic to the animals. In a next step, this association should be confirmed in other animal models.

### Pharmacokinetics and Pharmacodynamics of Zapnometinib in a Syrian Hamster Model

**FIGURE 2:**
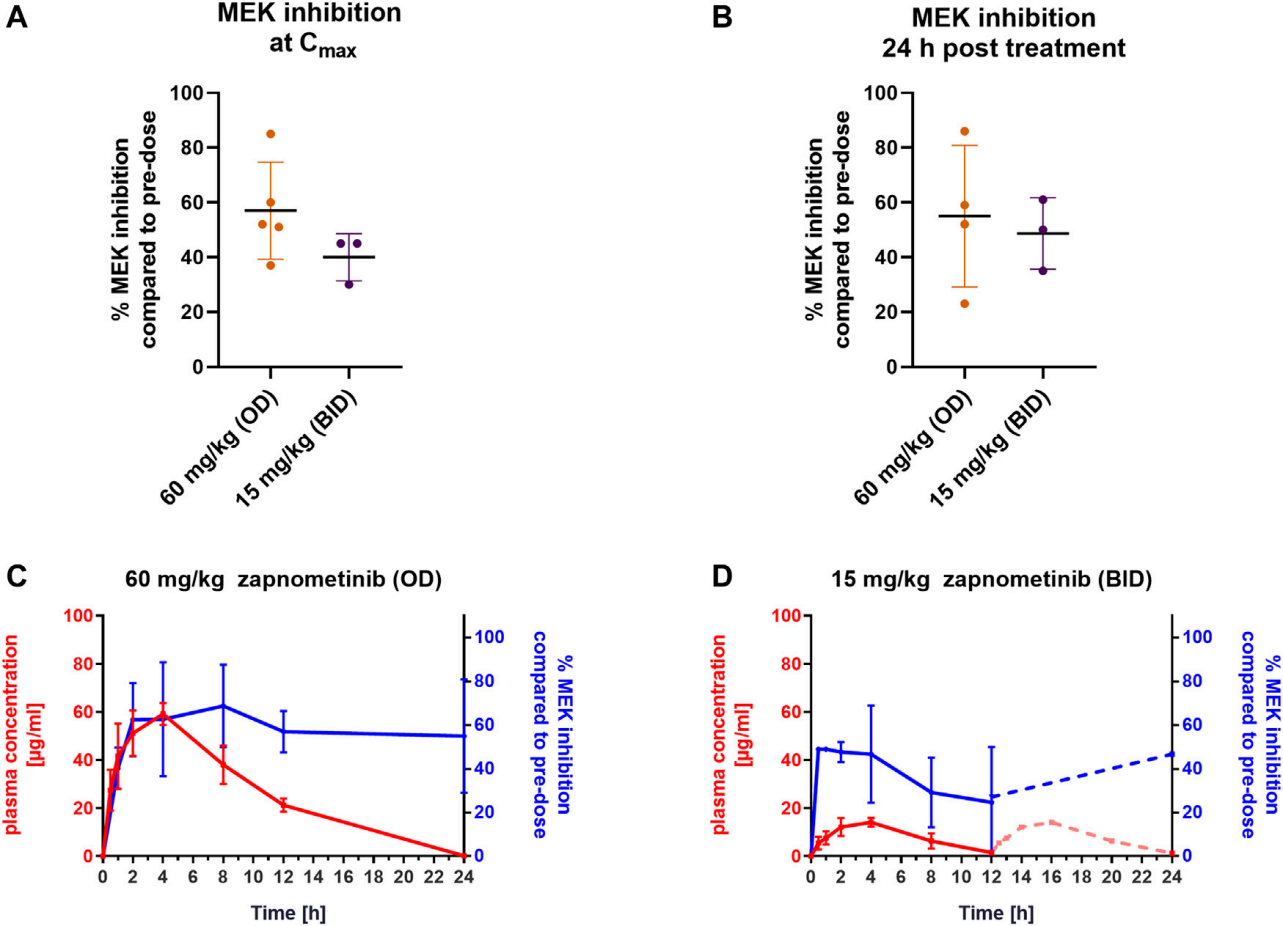
Pharmacokinetics and pharmacodynamics of zapnometinib in syrian hamsters. Syrian hamsters were treated with either a single dose of 60 mg/kg or twice daily with 15 mg/kg zapnometinib (*n* = 5 animals per group). The first treatment was given at *t* = 0 h and the second at 12 h post the first treatment (only for 15 mg/kg). Blood was collected at pre-dose, and 0.5, 1, 2, 4, 8, 12, and 24 h after the first treatment and analyzed for substance and ERK phosphorylation levels. Since no data were collected between 12 and 24 h the theoretical course of plasma concentration was calculated by adding the values from the first 12 h to the respective time points >12 h **(A,B)** MEK inhibition in PBMCs compared to pre-dose at (**A**) C_max_ (*t* = 4 h; *n*_(60 mg/kg)_ = 4; *n*_(15 mg/kg)_ = 3) or **(B)** 24 h post treatment (*n*_(60 mg/kg)_ = 4; *n*_(15 mg/kg)_ = 3). Data are presented as mean ± SD. **(C,D)** Plasma concentration (red line, left *y*-axis) of (**C**) 60 mg/kg and **(D)** 15 mg/kg of zapnometinib correlated with MEK inhibition (blue line, right *y*-axis). Dashed red line between 12 and 24 h shows extrapolated course of the plasma concentration and dashed blue line indicates that no values between 12 and 24 h were analyzed for MEK inhibition. Data are presented as mean ± SD (*n* = 5).

The Syrian hamster is a useful model to study SARS-CoV-2 infections (Imai et al., 2020). Since zapnometinib may also be suitable as a treatment option against SARS-CoV-2, we performed a pharmacokinetic and pharmacodynamic analysis in Syrian hamsters to determine the dose and exposure to zapnometinib required to inhibit MEK. The treatment of infected hamsters *via* oral gavage is not safe or practicable for both operators and the animals themselves. Thus, we tested whether a strawberry-flavoured zapnometinib formulation, which the animals accept voluntarily, would lead to reliable PK and PD data. This approach improves both animal welfare and more adequately simulates the clinical setting in paediatric use or in those unable to swallow pills. One group of animals was treated once with a dose of 60 mg/kg zapnometinib and the other group was treated twice with a dose of 15 mg/kg zapnometinib, with the second dose administered 12 h after the first dose. After a single oral administration of 60 mg/kg zapnometinib (Figure 2C; red line), a mean C_max_ of 61 ± 4 μg/ml after 3 ± 1 h and a mean AUC_0–24 h_ of 623 ± 87 μg/ml_ _h was reached (Table 2). In contrast, the 15 mg/kg zapnometinib (Figure 2D; red) reached a mean C_max_ of 15 ± 1 μg/ml after 3 ± 1 h, with a mean AUC_0–24 h_ of 121 ± 4 μg/ml∙h (Table 2).

**TABLE 2:** Summary of Pharmacokinetic Parameters in Syrian hamsters.

Dose (mg/kg) n = 5	C_max_ (µg/ml)	AUC_0–24 h_ (µg/ml*h)	T_max_ (h)
60 (OD)	61 ± 2	623 ± 87	3 ± 1
15 (BID)	15 ± 1	121 ± 4	3 ± 1

Values are reported as mean ± standard deviation (SD). n–number of subjects; C_max_, maximum observed plasma concentration; AUC, area under the concentration-time- curve; T_max_, time to maximum plasma concentration; OD, once daily; BID, bidaily.

Next, the level of MEK inhibition at C_max_ and 24 h post-treatment with both doses, 15 mg/kg (BID) and 60 mg/kg (OD) compared to pre-dose level were examined (Figures 2A,B). The dose of 60 mg/kg reached MEK inhibition of 57 ± 17% (*n* = 5) at a C_max_ of 61 ± 4 μg/ml. With the dose of 15 mg/kg, 40 ± 9% (*n* = 3) MEK inhibition was achieved with a C_max_ of 15 ± 1 μg/ml (Figures 2A,C). Even 24 h after zapnometinib administration, MEK inhibition was still observed at both doses (Figure 2B). In animals treated with 60 mg/kg zapnometinib, MEK inhibition of 55 ± 26% (*n* = 4) was still observed 24 h after treatment with a mean plasma concentration of 0.2 ± 0.6 μg/ml zapnometinib (Figure 2C). Animals treated twice with 15 mg/kg zapnometinib still showed 25 ± 15% MEK inhibition with a plasma concentration of 2 ± 0.1 μg/ml zapnometinib 12 h after the first treatment. After administration of the second dose at 12 h, the plasma concentration should increase again (theoretical course was calculated by adding the values from the first 12 h to the respective time points >12 h and is shown as dashed red line in the Figure), which is reflected by another increase in MEK inhibition to 43 ± 8% (*n* = 4) measured at 24 h with a mean zapnometinib plasma concentration of 1 ± 0.2 μg/ml (Figure 2D).

These results support the findings from the investigations in the mouse model that MEK is still inhibited even though zapnometinib is largely eliminated from plasma 24 h after treatment.

### Pharmacokinetics and Pharmacodynamics of Different Zapnometinib Formulations in Beagle Dogs

Different zapnometinib formulations were prepared to determine the pharmacokinetic profile of zapnometinib in beagle dogs. A capsule formulation, three different tablet formulations and a liquid formulation were orally administered to each of 10 beagle dogs. The administration of zapnometinib by either a total oral dose of 300 mg/animal/occasion (tablet/capsule) or of 30 mg/kg (liquid formulation) led to different pharmacokinetic profiles. The liquid formulation resulted in the highest C_max_ at 2 h after treatment (152 ± 25 μg/ml) and an AUC_0–24 h_ 962 ± 52 μg/ml*h (Table 3). Similar C_max_ values at 4 h after treatment were found for the tablet formulations ranging from 81 to 98 μg/ml and AUC_0–24_ _h_ values of 553–679 μg/ml*h (Table 3). The capsule formulation represented the lowest values for C_max_ (44 ± 16 μg/ml) at 4 h after treatment and AUC_0–24 h_ (352 ± 53 μg/ml∙h) (Table 3).

**TABLE 3:** Pharmacokinetic parameters after oral administration of zapnometinib in beagle dogs.

Formulation	Dosing route	C_max_ (µg/ml)	AUC_0–24 h_ (µg/ml*h)	T_max_ (h)	T_1/2_ (h)
1	Oral (capsule)	44 ± 16	352 ± 53	3 ± 0.4	5.0 ± 0.3
2	Oral (tablet)	93 ± 16	634 ± 39	4 ± 0.2	5.0 ± 0.5
3	Oral (tablet)	81 ± 23	553 ± 61	4 ± 0.02	3.7 ± 0.2
4	Oral (tablet)	98 ± 8	679 ± 36	4 ± 0.2	4.0 ± 0.3
5	Oral (liquid)	152 ± 25	962 ± 52	2 ± 0.1	4.2 ± 0.2

Values are reported as mean ± standard deviation (SD). **C**_**max**_—maximum observed plasma concentration; **AUC**, area under the concentration-time-curve. **T**_**max**_—time to maximum plasma concentration; **T**_**1/2**_—terminal half-life.

**FIGURE 3:**
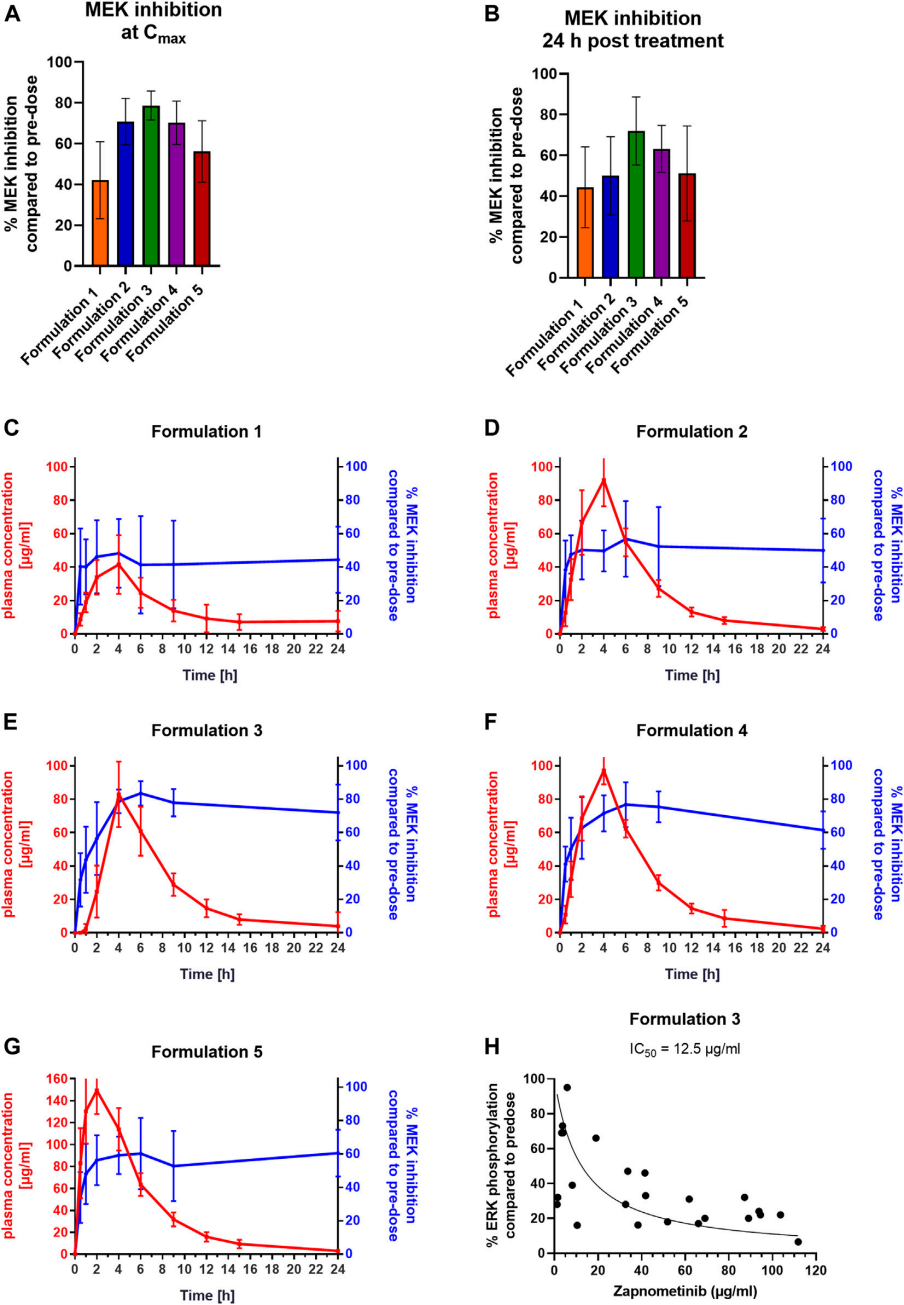
Pharmacokinetic Profiles and Pharmacodynamics of Different Zapnometinib Formulations in Beagle Dogs. Beagle dogs were treated with different formulations of zapnometinib. Blood was taken at pre-dose, 0.5, 1, 2, 4, 6, 9, 12, 15, and 24 h after treatment different timepoints and analyzed for plasma concentration and MEK inhibition. **(A,B)** PBMC samples of dogs **(A)** at the time of the highest plasma concentration C_max_ (*t* = 2 or 4 h; *n*_(formulation 1)_ = 9; *n*_(formulation 2)_ = 7; *n*_(formulation 3)_ = 10; *n*_(formulation 4)_ = 8; *n*_(formulation 5)_ = 10) or **(B)** 24 h post treatment (*n*_(formulation 1)_ = 8; *n*_(formulation 2)_ = 10; *n*_(formulation 3)_ = 10; *n*_(formulation 4)_ = 10; *n*_(formulation 5)_ = 10) were analyzed with Wes^TM^ for MEK inhibition levels and compared to pre-dose. Data are presented as mean ± SD. **(C–G)** Plasma concentration (red line, left *y*-axis) was plotted with MEK inhibition (blue line, right *y*-axis). Data are presented as mean ± SD (*n* = 10). **(H)** Relationship of zapnometinib concentration and target suppression (reduction of ERK phosphorylation) until reaching C_max_ is shown as a non-linear regression of ERK phosphorylation. The individual points represent individual values of ERK phosphorylation in dogs treated with zapnometinib.

Next, the level of MEK inhibition in the PBMCs of the Beagle dogs at C_max_ and at 24 h post treatment for the five different formulations was investigated (Figures 3A,B; Table 4). The strongest MEK inhibition was achieved with formulation 3 with 81 ± 23 μg/ml zapnometinib at C_max_ leading to an MEK inhibition of 79 ± 7% (*n* = 10) (Figure 3A). Comparable MEK inhibition values of 71 ± 11% (formulation 2, *n* = 7) and 70 ± 11% (formulation 4, *n* = 8) were reached with 93 ± 16 μg/ml (formulation 2) and with 98 ± 8 μg/ml zapnometinib (formulation 4) at C_max_. The weakest MEK inhibition was obtained with formulation 1 with 44 ± 16 μg/ml zapnometinib at C_max_ leading to 42 ± 19% MEK inhibition (*n* = 10). Formulation 5 achieved the highest plasma concentration of 152 ± 25 μg/ml zapnometinib, but only a moderate MEK inhibition of 56 ± 15% (*n* = 10). At 24 h after a single administration of zapnometinib, MEK inhibition was still observed for all formulations (Figure 3B). Formulation 3 achieved with 79 ± 17% (*n* = 10) the strongest MEK inhibition with a plasma concentration of 4 ± 8 μg/ml zapnometinib. Formulation 1 reached 44 ± 20% MEK inhibition with 6 ± 6 μg/ml zapnometinib. A mean MEK inhibition of 63 ± 12% could be obtained with 2 ± 2 μg/ml zapnometinib (formulation 4). Similar levels of 50 ± 19% (formulation 2) and 51 ± 23% (formulation 5) could be reached with mean plasma concentration of 3 ± 1 μg/ml (formulation 2) and 3 ± 1 μg/ml (formulation 5). When pharmacokinetic (red line) and pharmacodynamic parameters (blue line) were correlated, it was obvious that comparable with mice and hamsters, MEK inhibition in dogs was still present when zapnometinib was diminished from plasma at 24 h after treatment (Figures 3C–G). The concentrations of zapnometinib measured until C_max_ is reached can be correlated with the effect of MEK inhibition at the corresponding timepoints. This allows for creation of a dose-response curve and to calculate an IC_50_ of 12.5 μg/ml, the concentration of zapnometinib that is required to reduce ERK phosphorylation by 50% compared to pre-dose (Figure 3H).

**TABLE 4:** Association of PK and PD parameters of zapnometinib formulations in dogs.

Formulation	Dosing route	C_max_ (µg/ml)	MEK inhibition at C_max_ (%)	C_24 h_ (µg/ml)	MEK inhibition at 24 h (%)
1	Oral (capsule)	44 ± 16	42 ± 19	6 ± 6	44 ± 20
2	Oral (tablet)	93 ± 16	71 ± 11	3 ± 1	50 ± 19
3	Oral (tablet)	81 ± 23	79 ± 7	4 ± 8	79 ± 17
4	Oral (tablet)	98 ± 8	70 ± 11	2 ± 2	63 ± 12
5	Oral (liquid)	152 ± 25	56 ± 15	3 ± 1	51 ± 23

Values are reported as mean ± standard deviation (SD). **C**_**max**_—maximum observed plasma concentration; **C**_**24** **h**_–observed plasma concentration at *t* = 24 h.

These data show that in dogs, as in rodents, MEK inhibition was proportional to C_max_, and was maintained despite elimination of the substance from plasma. The general similarity in the results suggested extension to human studies.

### Pharmacokinetics and Pharmacodynamics of Zapnometinib in Healthy Human Volunteers

To test whether the PK and PD findings from different animal species translate to humans, we conducted a phase 1 clinical trial in SAD format with 10 healthy volunteers each treated with 100, 300, 600 or 900 mg zapnometinib.

**FIGURE 4:**
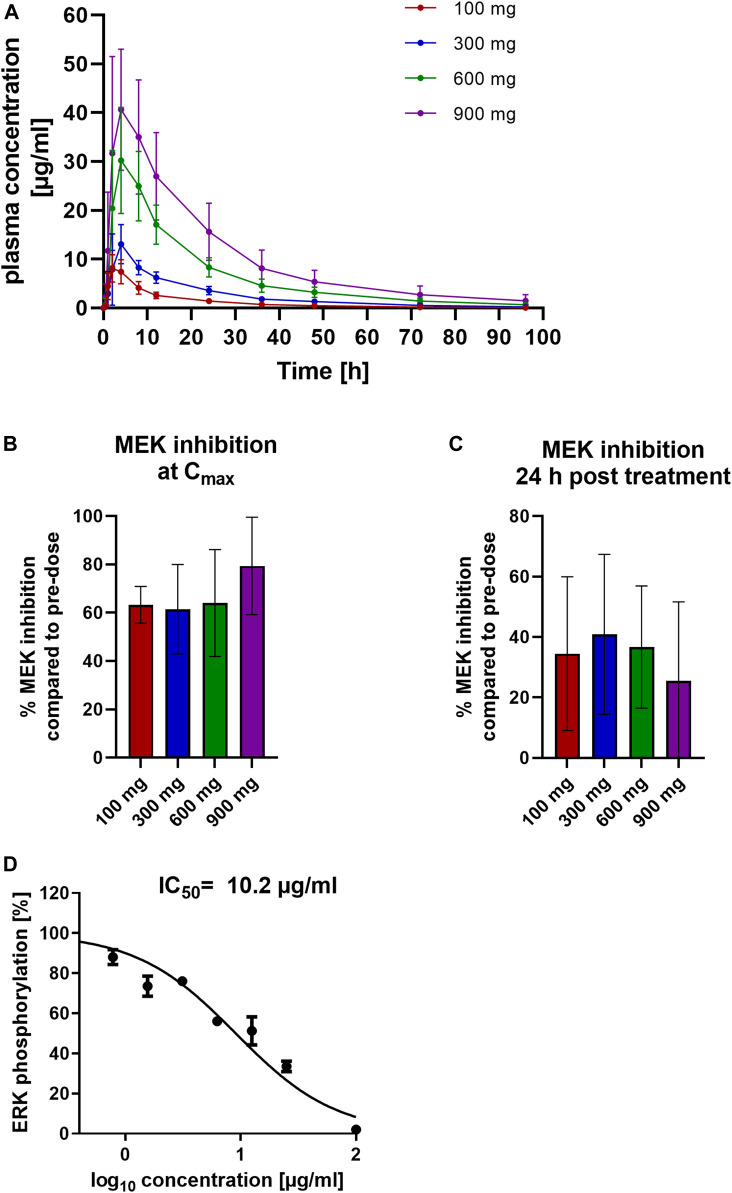
Pharmacokinetic and pharmacodynamic assessment of zapnometinib in humans. Healthy volunteers were randomized to four cohorts for a single ascending dose in a phase 1 clinical trial and were treated with either 100, 300, 600 or 900 mg zapnometinib. Blood was taken 0.5, 1, 2, 4, 8, 12, and 24 h later and analyzed for plasma concentration and MEK inhibition. Additionally, blood was taken at 36, 48, 72, and 96 h after treatment for the MAD part. **(A)** Plasma concentrations following a single dose of 100, 300, 600 and 900 mg zapnometinib. Data are presented as mean ± SD (*n* = 8). **(B)** Mean MEK inhibition at C_max_ compared to pre-dose analyzed by ERK phosphorylation with Wes^TM^ in PBMCs at the highest zapnometinib plasma concentration (C_max,_ *t* = 2–4 h; *n*_(100 mg)_ = 7; *n*_(300 mg)_ = 7; *n*_(600 mg)_ = 6; *n*_(900 mg)_ = 5) and (**C**) 24 h post treatment (*n*_(100 mg)_ = 8; *n*_(300 mg)_ = 8; *n*_(600 mg)_ = 3; *n*_(900 mg)_ = 3). (**D**)Human whole blood was treated with different concentration of zapnometinib (100 μg/ml to 0 μg/ml) for 1 h at 37°C and 5% CO_2_. PBMCs were isolated and analysed for MEK inhibition. Determination of IC_50_ value by plotting %ERK phosphorylation against log_10_ of zapnometinib concentration, using nonlinear regression fit in GraphPad Prism. Data are presented as mean ± SD (*n* = 3 blood donors).

After a single oral dose of zapnometinib in tablet form, the mean C_max_ was reached 2 h post dose for 100 mg zapnometinib and 4 h post dose for 300, 600 and 900 mg. A maximum plasma concentration of 9 ± 2 μg/ml was achieved with a treatment dose of 100 mg zapnometinib, 13 ± 4 μg/ml with 300 mg, 35 ± 7 μg/ml with 600 mg, and 46 ± 16 μg/ml with 900 mg zapnometinib. After C_max_, the zapnometinib plasma concentrations declined in a multi-phasic manner and in parallel for all doses (Figure 4A; Table 5). Analyses of MEK inhibition at C_max_ revealed that with a C_max_ of 46 ± 16 μg/ml the strongest inhibition of 79 ± 20% was achieved with the highest single dose of 900 mg zapnometinib (Figure 4B; Table 6). Treatment with the other doses (100, 300, and 600 mg) resulted in MEK inhibition of approximately 60% (100 mg: 63 ± 8%, 300 mg: 61 ± 18%, 600 mg: 64 ± 22%) (Figure 4B). Even 24 h after zapnometinib treatment, MEK inhibition of 35 ± 25% with a zapnometinib plasma concentration of C_24 h_ = 1 ± 0.4 μg/ml (100 mg), 41 ± 26% with C_24 h_ = 4 ± 0.8 μg/ml (300 mg), 37 ± 20% with C_24 h_ = 8 ± 2 μg/ml (600 mg) and 26 ± 26% (900 mg) with C_24 h_ = 16 ± 6 μg/ml were found (Figure 4C; Table 6).

**TABLE 5:** Summary of zapnometinib pharmacokinetic parameters for SAD study in healthy volunteers.

Dose *n* = 8	C_max_ (µg/ml)	AUC_0–24 h_ (µg/ml*h)	AUC_0-t_ (µg/ml*h)	T_max_ (h)	T_1/2_ (h)
100 mg	9 ± 2	84 ± 9	115 ± 10	2* (2–4)**	19.6 ± 5.3
300 mg	13 ± 4	157 ± 16	241 ± 18	4* (2–4)**	19.8 ± 5.8
600 mg	35 ± 7	411 ± 44	617 ± 51	4* (4–8)**	21.9 ± 6.8
900 mg	46 ± 16	629 ± 83	1,001 ± 107	4* (2–8)**	23.7 ± 8.2

Values are reported as mean ± standard deviation (SD) except median (min-max) for T_max_. ***n***—number of subjects; **C**_**max**_—maximum observed plasma concentration; **AUC**, area under the concentration-time- curve; **T**_**max**_—time to maximum plasma concentration; **T**_**1/2**_—terminal half-life; * mean value; ** range.

**TABLE 6:** Association of PK and PD parameters of zapnometinib in human.

Dose n = 8	C_max_ (µg/ml)	MEK inhibition at C_max_ (%)	C_24 h_ (µg/ml)	MEK inhibition at 24 h (%)
100 mg	9 ± 2	63 ± 8	1 ± 0.4	35 ± 25
300 mg	13 ± 4	61 ± 18	4 ± 0.8	41 ± 26
600 mg	35 ± 7	64 ± 22	8 ± 2	37 ± 20
900 mg	46 ± 16	79 ± 20	16 ± 6	26 ± 26

Values are reported as mean ± standard deviation (SD). **n**—number of subjects; **C**_**max**_—maximum observed plasma concentration; **C**_**24** **h**_–observed plasma concentration at *t* = 24 h.

In a separate experiment, we treated whole blood of three healthy volunteers with different concentrations of zapnometinib (in the range of 100 μg/ml to 0 μg/ml), then isolated PBMCs and measured apparent MEK activity via ERK phosphorylation. In line with the IC_50_ value observed in dogs, at least 10 μg/ml of zapnometinib were required to reduce ERK phosphorylation by 50% (Figure 4D).

In summary, the results show that up to 80% MEK inhibition in PBMCs was observed after zapnometinib treatment regardless of species. 24 h after dosing, when almost all zapnometinib was eliminated from the plasma, MEK inhibition of up to 50% was maintained.

## Discussion

Zapnometinib is a potent and selective MEK1/2 inhibitor that is currently in phase 2 clinical trials as a potential agent against COVID-19 (NCT04776044). Due to its mode of action, it could also be used against other viral infections, like influenza (Pleschka, 2008; Pinto et al., 2011; Laure et al., 2020). Since zapnometinib targets a host cell factor, investigation of the dose response relationship is useful to better understand and predict the pharmacology of the antiviral effect.

The aim of this study was to investigate which dose of zapnometinib will lead to pharmacodynamically active concentrations in mice, hamsters, dogs, and humans. In addition, the study provided data on the duration of apparent MEK inhibition, and the effects of dose and formulation. These observations lead to questions on the degree of inhibition required for antiviral effects, the turnover of pERK, the mechanism for sustained MEK inhibition, the relevant tissues, and compartments to measure and the optimal dose regime for antiviral and anti-inflammatory effects.

To measure drug interaction with the target, we used PBMCs as a model for lung tissue given that these can be easily obtained from healthy volunteers and patients. Since COVID-19 infection and also influenza virus infection occurs mainly in the lungs, lung cells would be more relevant. However, it is not possible to take lung samples from a living organism. Since blood cells are easy to collect and the signaling pathway is also activated in these cells by virus infection and is involved in many immune response processes, we use PBMCs. In follow-up studies, we will relate lung to PBMC values to understand how this parameter translate between peripheral blood and lung tissue. We found that zapnometinib leads to up to 80% MEK inhibition in PBMCs at doses that were well tolerated for the respective species. MEK inhibition in PBMCs was also detectable 24 h after treatment, when zapnometinib was mostly eliminated from plasma.

In mice, a 60%–80% MEK inhibition in PBMCs was sufficient to reduce influenza virus titer in lungs by >90%. Treatment of influenza virus infected mice with 25 mg/kg zapnometinib BID leads to a favorable pharmacokinetic profile and to an inhibition of MEK activity in PBMCs between 57%–80%. However, given the practical limits of sampling from infected animals over time, a detailed time course is not feasible and reduction of ERK phosphorylation, and thus MEK inhibition may also have been transiently higher than 80%. Although no substance was detectable in plasma after 24 h, MEK inhibition (measured as reduction of ERK phosphorylation) in PBMCs remained at 66%. The cause of the sustained effect on pERK levels remains to be clarified, however, based on other factors and the relationship with C_max_, we hypothesize that it indicates, inter alia, a slow off-rate. Alternatively, accumulation of substance to PBMCs or changes in regulation of the cascade may lead to this effect and we are examining the possible mechanisms of sustained levels of pERK after drug elimination.

Since the Syrian hamster represents a very suitable model to study COVID-19 (Imai et al., 2020), this animal was used to investigate zapnometinib mediated MEK inhibition. In animals treated with 60 mg/kg zapnometinib, MEK inhibition of approximately 63% was achieved at a C_max_ of 61 μg/ml. Treatment with 15 mg/kg (BID) resulted in a C_max_ of 15 μg/ml and MEK inhibition of less than 50%. After 24 h, MEK inhibition levels were still relatively high at 55% (60 mg/kg) and 49% (15 mg/kg; BID), despite the low zapnometinib concentration in plasma. Based on these data, higher doses of zapnometinib are recommended in a Syrian hamster SARS-CoV-2 infection model to achieve a constant MEK inhibition of more than 50%.

In Beagle dogs the different formulations applied resulted in different pharmacokinetic profiles, leading to C_max_ levels ranging from 44–152 μg/ml. The inhibition of MEK and the resulting reduction in ERK phosphorylation was 40%–80% at the time of C_max_. Interestingly, we did not find the strongest MEK inhibition in PBMCs at the highest C_max_ value. Therefore, we hypothesize that the maximum achievable MEK inhibition of 80% has already been reached and that higher inhibition levels cannot be achieved even with a higher plasma concentration of zapnometinib. To better understand this observation, we are examining the kinetics of MEK inhibition onset vs. time to determine whether there is a lag between T_max_ and maximal MEK inhibition. Again, after 24 h MEK inhibition levels were still between 44%–72%, although zapnometinib concentration was low in plasma (2–8 μg/ml). The difference between formulation 3 and 5 in terms of MEK inhibition may be due to the faster plasma levels achieved. Formulation 5 reached much faster higher plasma levels than formulation 3, but zapnometinib has high plasma binding. Therefore, we assume that not all of the compound reached the cell. And since formulation 5 reached C_max_ faster, we suspect that it is too fast for zapnometinib to inhibit MEK in all cells, this could explain higher levels seen in the green bars (Figures 3A,B).

Comparing the zapnometinib concentrations in the plasma up to C_max_ with the corresponding MEK inhibition in PBMCs, we were able to determine an IC_50_ of 12.5 μg/ml zapnometinib for formulation 3. For formulation 1, and 2, no dose response curve could be generated for the calculation of an IC_50_, as not more than 50% MEK inhibition could be reached in the increase of plasma concentration until reaching C_max_. And for formulation 5, no dose-response curve could be generated due to the too fast increasing plasma concentration. A slightly lower IC_50_ value of 10 μg/ml could be observed in human PBMCs, supporting the simplified conclusion that a zapnometinib plasma concentration of approximately 10 μg/ml is required to achieve 50% MEK inhibition.

In humans, roughly 60% MEK inhibition was detectable when healthy volunteers were treated with 100–600 mg zapnometinib, treatment with 900 mg resulted in MEK inhibition of 83%. The observation that MEK is still partially inhibited 24 h after treatment despite reduced plasma levels was also apparent in human subjects. This may suggest that a formulation focused on C_max_ may be preferable to one driven by AUC in terms of the period of pERK suppression.

The degree to which constant pERK suppression is required is a key question in dose selection. While permanent activation of the Raf/MEK/ERK signaling is common in cancer, the activation during virus infection is transient. Thus, while oncology therapy paradigms require near complete target occupancy for long periods of time, viral therapy may be adequate with partial inhibition over shorter periods. In oncology, high levels of sustained target occupancy require regular high doses which leads to issues of patient tolerance. These tolerance effects would be counterproductive in the infection setting where immune function should be maintained. Thus, the observation that 50%–80% MEK function reduction, as measured by apparent pERK/ERK ratios, is sufficient to block viral assembly, suggests that lower doses are adequate for beneficial effects in the antiviral setting.

However, measuring these pharmacodynamic parameters is more straightforward in cancer than in infection because of the availability of biopsy material in cancer that cannot be obtained easily from infected patients in a phase 1 or 2 study.

Similar to our results, in a study with the MEK inhibitor trametinib, the ability to inhibit ERK phosphorylation was also investigated *in vivo* and the efficacy was confirmed in a mouse tumor xenograft model. This study demonstrated that treatment with trametinib results in sustained inhibition of ERK phosphorylation which is consistent with the PK profile of the drug. These results confirm our observations that a reduction in ERK phosphorylation was still evident after 24 h with a low plasma concentration (Gilmartin et al., 2011). In another study, the highest MEK inhibition was also achieved with the highest plasma concentration of the inhibitor after a single application of the MEK inhibitor RG7204 in a LOX melanoma tumor xenograft model. Even with a decrease in plasma concentration, the inhibition remained at a relatively constant value up to 8 h post dose (Yang et al., 2010). This is in line with our observations that the maximal effect is found at the highest plasma concentration of the drug and supports our data on the association between C_max_ and inhibition of ERK phosphorylation.

The method used to investigate drug-target interaction *in vivo* should be considered when assessing these results (Simon et al., 2013; Main and Zhang, 2020). In our study, we used a quantitative capillary electrophoresis to detect pERK/ERK. Other studies used semi-quantitative methods such as immunohistochemistry, Western Blot or a quantitative duplex ELISA to investigate the reduction of ERK phosphorylation (Lorusso et al., 2005; Yang et al., 2010; Gilmartin et al., 2011).

A key question is whether 80% MEK inhibition represents the highest feasible value as found in mice and dogs or whether this value could be increased with higher zapnometinib dosage. We were not able to further increase the dose of zapnometinib in the human study above 900 mg, due to a shortage of formulated active pharmaceutical ingredients (API). A further PK/PD study will be conducted with doses higher than 900 mg to address this question. In humans, we could not measure the concentration of zapnometinib in the lung, the main target organ of the drug in respiratory infection. We are currently planning another preclinical study in animal models to get a better insight into the correlation of plasma vs. lung concentration ratio.

In summary, we could show the interrelationships of PK and PD for the MEK inhibitor zapnometinib in three relevant animal species and in healthy human volunteers. Independent of the species, we did not observe MEK inhibition of more than 80% but sustained activity to 24 h after a single dose when most substance was eliminated from the plasma. In the mouse model, we were able to show a correlation of PK/PD data with antiviral efficacy, where 60%–80% MEK inhibition was sufficient to reduce viral titers in the lung by more than 90%. These results provide a better understanding of the zapnometinib target engagement and will influence the design of further clinical trials.

## Data Availability

The original contributions presented in the study are included in the article/Supplementary Material, further inquiries can be directed to the corresponding author.
