# Supplementary material for: Pharmacokinetics, Pharmacodynamics and Antiviral Efficacy of the MEK Inhibitor Zapnometinib in Animal Models and in Humans
Source: Front Pharmacol. 2022 Jun 15;13:893635. doi: 10.3389/fphar.2022.893635 (PMC9240354; doi:10.3389/fphar.2022.893635)
Supplement: Supplementary file 1 [file DataSheet1.docx]

Supplementary Material

# Determination of Phosphorylation Levels in Human PBMCs

PBMCs of 30 ml human blood were isolated using SepMate^TM^ tubes (Stemcell Technologies, Vancouver, Canada) by density gradient centrifugation according to manufacturer’s instructions. The PBMCs were then cultured in the plasma and treated with 50 µg/ml zapnometinib or DMSO for 1 h at 37°C and 5% CO_2_. Afterwards, the PBMCs were stimulated with 400 nM PMA (Sigma-Aldrich, St. Louis, Missouri, USA) for 30 minutes at 37°C and 5% CO_2_. After stimulation, the cells were washed twice with PBS and centrifuged 8 minutes at 300 x g at RT. The cells were then lysed in 1x RIPA buffer and analyzed with Wes™ for phosphorylation of ERK and MEK using specific antibodies as described in the Pharmacodynamic assessment to determine level of MEK inhibition section. The following antibodies were used: Phospho-MEK1/2 (Ser217/221) antibody (Cell Signaling Technology, Cat #9121) and MEK1/2 antibody (Cell Signaling Technology, Cat#9122) in a 1:10 dilution in Antibody Diluent (ProteinSimple^®^), phospho ERK1/2 (Phospho-p44/42 MAPK (Erk1/2) (Thr202/Tyr204) (D13.14.4E) XP^®^ Rabbit mAb, Cell Signaling Technology, Cat# 4370) and ERK1/2 (p44/42 MAPK (Erk1/2) (137F5) Rabbit mAb, Cell Signaling Technology, Cat# 4695).

# Supplementary Figure


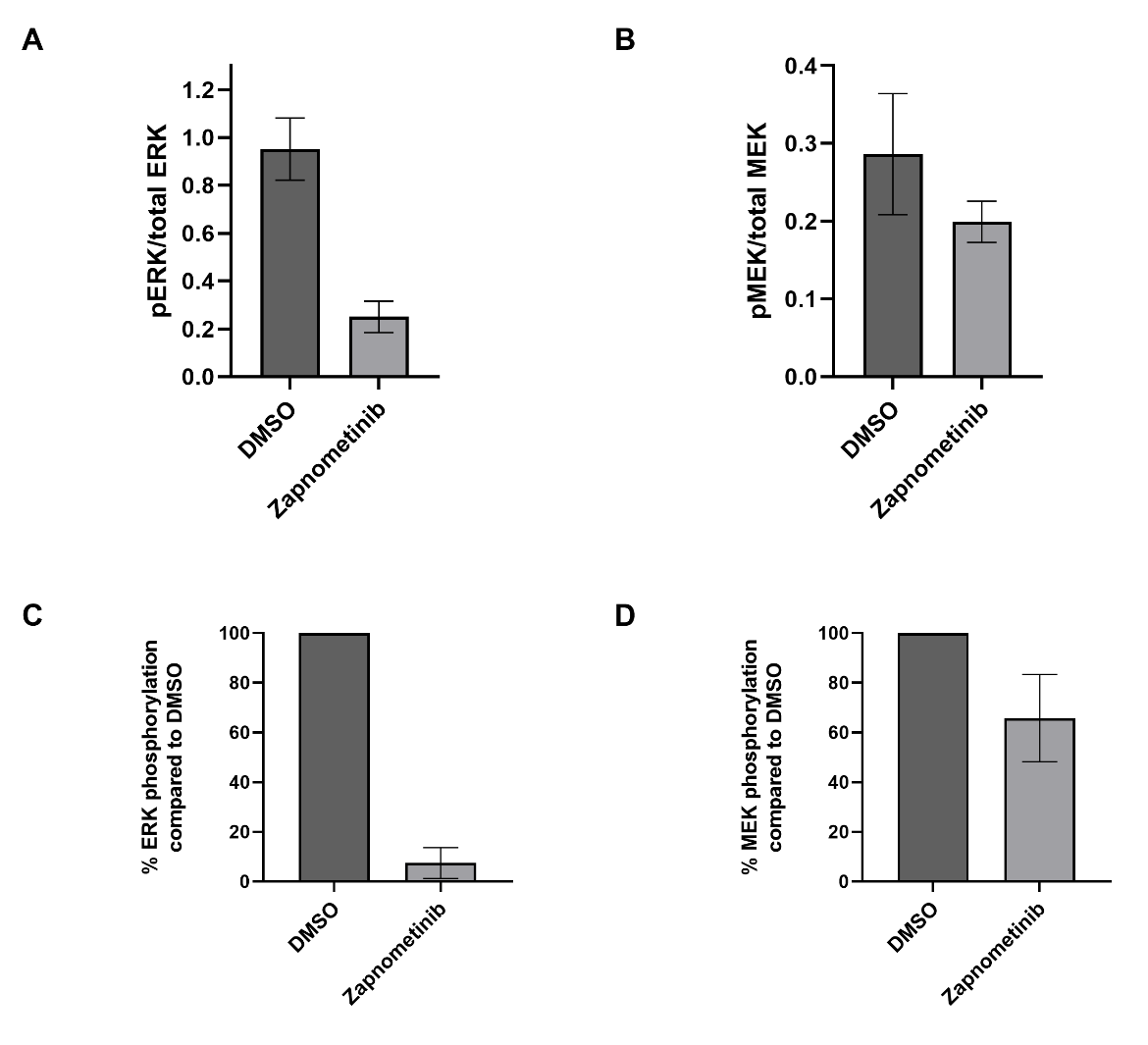


**Supplementary Figure 1.** **Effect of zapnometinib on the phosphorylation of MEK compared with ERK in human PBMCs.** PBMCs of healthy human volunteers were treated for 1 h with 50 µg/ml zapnometinib or DMSO as solvent control and subsequently stimulated for 30 minutes with 400 nM PMA. Cell lysates of the PBMCs were then analyzed with Wes™ for phosphorylated and total protein, and the ratio was used to calculate the phosphorylation levels. Mean ratio of phosphorylated protein to total protein of pERK/ERK (**A**) and pMEK/MEK (**B**). Mean % phosphorylation levels of ERK (**C**) and MEK (**D**) in human PBMCs after treatment with zapnometinib compared to DMSO, which was set as 100% phosphorylation. Bars show mean phosphorylation level ± SD (n = 6).

# Supplementary Table 1 Summary of adverse events in humans

| **System Organ Class**  Adverse event, n (%) | **Placebo**  **n = 8** | **100 mg**  **n = 8** | **300 mg**  **n = 8** | **600 mg**  **n = 8** | **900 mg**  **n = 8** |
| --- | --- | --- | --- | --- | --- |
| **Gastrointestinal Disorders** | **0** | **0** | **0** | **0** | **2 (25.0)** |
| Abdominal pain | 0 | 0 | 0 | 0 | 1 (12.5) |
| Diarrhea | 0 | 0 | 0 | 0 | 1 (12.5) |
| **Infections and Infestations** | **0** | **0** | **1 (12.5)** | **0** | **0** |
| Nasopharyngitis | 0 | 0 | 1 (12.5) | 0 | 0 |
| **Investigations** | **0** | **0** | **1 (12.5)** | **0** | **0** |
| Hepatic enzyme increase | 0 | 0 | 1 (12.5) | 0 | 0 |
| **Musculoskeletal and Connective Tissue Disorders** | **0** | **0** | **0** | **2 (25.0)** | **0** |
| Back pain | 0 | 0 | 1 (12.5) | 0 | 0 |
| Neck pain | 0 | 0 | 1 (12.5) | 0 | 0 |
| **Nervous System Disorders** | **0** | **0** | **3 (37.5)** | **1 (12.5)** | **1 (12.5)** |
| Dizziness | 0 | 0 | 0 | 0 | 0 |
| Headache | 0 | 0 | 3 (37.5) | 1 (12.5) | 1 (12.5) |
| **Respiratory, thoracic and Mediastinal Disorders** | **0** | **0** | **1 (12.5)** | **0** | **1 (12.5)** |
| Rhinorrhea | 0 | 0 | 1 (12.5) | 0 | 0 |
| Throat irritation | 0 | 0 | 0 | 0 | 1 (12.5) |
| **Skin and Subcutaneous Disorders** | **0** | **0** | **1 (12.5)** | **0** | **1 (12.5)** |
| Erythema | 0 | 0 | 1 (12.5) | 0 | 1 (12.5) |
